# Antidepressant effect of recombinant NT4-NAP/AAV on social isolated mice through intranasal route

**DOI:** 10.18632/oncotarget.14356

**Published:** 2016-12-29

**Authors:** Fei Liu, You-ping Liu, Gang Lei, Peng Liu, Zheng Chu, Cheng-ge Gao, Yong-hui Dang

**Affiliations:** ^1^ College of Medicine & Forensics, Xi’an Jiaotong University Health Science Center, Xi’an, 710061, Shaanxi, PR China; ^2^ Clinical Research Center of Shaanxi Province for Dental and Maxillofacial Diseases, College of Stomatology, Xi'an Jiaotong University, Xi'an, 710004, PR China; ^3^ Sun Yat-sen University Cancer Center, Guangzhou, 510060, PR China; ^4^ Department of Psychiatry, First Affiliated Hospital of Xi’an Jiaotong University Health Science Center, Xi’an, 710061, Shaanxi, PR China; ^5^ Key Laboratory of the Health Ministry for Forensic Medicine, Xi'an Jiaotong University Health Science Center, Xi’an 710061, Shaanxi, PR China; ^6^ Key Laboratory of Forensic Medicine of Shaanxi Province, Xi’an Jiaotong University Health Science Center, Xi’an 710061, Shaanxi, PR China

**Keywords:** social isolation, depression, NT4-NAP/AAV, intranasal administration, antidepressant

## Abstract

The purpose of the present study was to observe the depression-like behavior induced by social isolation; detect the antidepressant effect of a recombinant adeno-associated virus (AAV) expressing NAP on social isolation mice by intranasal delivery. After construction of NT4-NAP/AAV, expression of NAP was confirmed *in vitro*. 3-week-old C57/BL mice were bred individually in cages as social isolation-rearing. Six weeks later, the first subset of mice underwent behavioral tests and western blot; the second was for enzyme-linked immunosorbent assay. NT4-NAP/AAV was delivered quaque die by nasal administration for consecutive 10 days before behavioral test. Several depression-like behaviors were observed in social isolation mice, including decreased relative sucrose preference, longer immobility time in forced swimming test, lower plasma corticosterone and decreased brain-derived neurotrophic factor in hippocampus. Thus, social isolation procedure appears to be an animal model of depression with good face and construct validity. What's more, the antidepressant effect in social isolation-rearing mice was observed after intranasal administration of NT4-NAP/AAV, suggesting that this might be a promising therapeutic strategy for depressive disorder.

## INTRODUCTION

Major depressive disorder (MDD) is a heterogeneous illness that manifests in a variety of symptom sets including depressed mood, insomnia or hypersomnia, feelings of worthlessness or excessive or inappropriate guilt, markedly diminished interest or pleasure and recurrent thoughts of death which bring a lot of troubles to the victims [1]. The life time prevalence of depressive disorder ranging between 15% and 20%[2], with an onset at different points in life and lasts for a varying amount of time. Except for the genetic factors, environmental challenges appear to have a crucial influence on the disease, either increasing or decreasing the disease risks of depressive disorder patients [3]. A wealth of evidence showed that adverse experiences during early life, such as maternal separation (MS) or social isolation (SI), could lead to the changes of brain development, neurochemical effects and adult behaviors in rodents [4–6], which might be related to clinical core symptoms in patients with some certain neuropsychiatric disorders. The abnormal behaviors induced by SI-rearing include aggressive behavior [7], anxiety-like behavior [8], impairment of prepulse inhibition [9] and cognitive deficits [7]. Although some researchers failed to detect the obviously depression-like behavior in SI mice [10], it has been increasingly recognized nowadays [6, 11, 12].

Dysregulation of hypothalamic-pituitary-adrenal (HPA) axis, monoamine and hippocampal BDNF insufficient were three cardinal hypotheses of pathogenesis of depression. Based on the hypotheses of pathogenesis, psychiatrists and neuroscientists have devoted themselves to the treatment of depression for years, but the therapeutic effect is less satisfactory than expected. In clinic, a proportion of patients are not sensitive to the anti-depressants currently in use. The effective cases are always accompanied with a chronic administration, delayed onset of clinical efficacy and unavoidable side-effects [13, 14].

Recently, new therapeutic drugs and methods for neuropsychitric disorder emerges one after another, and NAP (Asn-Ala-Pro-Val-Ser-Ile-Pro-Gln,NAPVSIPQ) is one of the most outstanding representatives. It is an eight-amino-acid peptide derived from activity dependent neuroprotective protein (ADNP), was shown to have a brain bioavailability and neuroprotective effects in a wide variety of neurological disorders [15, 16]. *In vivo* studies, NAP has been readily delivered to brain by subcutaneous, intranasal [17], iv and ip routes [15]. The neuroprotection and cognitive enhancement by NAP exhibited not only in disease models, but also in healthy animals [16]. However, the reasearch of NAP on depressive disorder is still at an early stage. How to maintain the effective concentration of NAP in brain and make cost down are issues that need to be addressed urgently.

The gene therapy approach mediated by recombinant adeno-associated virus (AAV) is getting more and more popular because it could infect and transduce postmitotic cells like neurons [18], provoke minimal inflammatory response, and produce stable, long-term (at least one year), episomal expression of viral transgene by a few times’ treatment (usually once)[18–20]. It has been applied successfully in many studies to transduce central nervous system and other tissues [18–20]. Peripheral administration of AAV through nose is an non-invasive route which could avoid the adverse influence of central administration, and take full advantages of AAV vector [21, 22]. In our previous study, we have constructed a recombinant AAV which could express neurotrophin 4 (NT4) and NAP in brain by intranasal delivery [22]. Anti-depression effects on the normal female C57BL/6 mice were observed in forced swimming test after 10 days’ administration [22].

Thus, there are two objectives in the current study. Firstly, to detect whether SI-rearing would induce the depression-like behaviors in mice. Secondly, to observe the anti-depression effect of recombinant NT4-NAP/AAV on SI mice by intranasal delivery.

## RESULTS

### The construction and packaging of NT4-NAP/AAV

**Figure 1:**
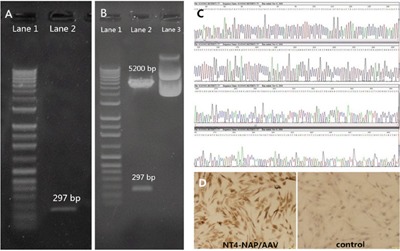
Construction and identification of NT4-NAP/AAV **A, B**. Identification of the NAP cDNA with the added restriction enzyme sites with EcoR. A: Lane 1 DNA Marker, Lane 2 PCR product. B: Lane 1 DNA Marker, Lane 2 Recombinant Plasmid pGEM-T Easy after restriction enzyme reaction, Lane 3 Recombinant Plasmid pGEM-TEasy. **C**. Sanger sequencing. The produced sequence of the fusion gene, NT4-NAP, was identical as the designed one. **D**. Immunocytochemistry result of PC12 cells. Compared with the control ones, the infected PC12 cells showed high expression of NT4-NAP.

Every step of NT4-NAP fusion gene construction was qualified by specific restriction enzyme reactions and agarose gel electrophoresis (AGE). After BamH I and Xho I sites were added in the NAP cDNA by PCR, the PCR product was identified with digesting by restriction enzyme EcoR I followed by AGE. Meanwhile, a 297 bp fragment can be observed when the recombinant plasmid pSSCMV/NT4-NAP was digested by EcoR I followed by AGE (Figure 1-A, 1B). The fusion gene, NT4-NAP, was identified using the Sanger sequencing, and the produced sequence was identical as the designed one (Figure 1-C). Immunocytochemistry results showed that expression of NAP can be observed in PC12 cells infected with NT4-NAP/AAV, while there was no expression in the control group (Figure 1-D).

### Effect of NT4-NAP/AAV on the OFT

**Figure 2:**
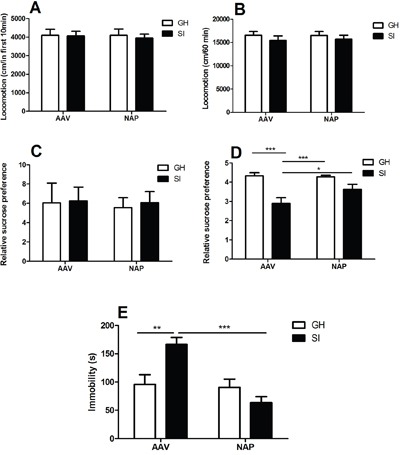
The behavioral tests **A-B**. Open field test. No differences were evoked by NT4-NAP/AAV administration in the first 10 minutes (A) and the total 60 minutes (B). **C-D**. Sucrose consumption test. No statistical differences among the four groups at baseline (C). After conditional rearing, the SI mice showed decreased relative sucrose preference compared with GH mice in AAV group (P< 0.001), and the NT4-NAP/AAV treatment showed an significantly enhanced relative sucrose preference in SI mice (P< 0.05)(D). **E**. Forced swimming test. SI mice showed longer immobility time compared with GH mice in AAV group (P< 0.01), and the NT4-NAP/AAV treatment showed an anti-depression efficacy in SI mice (P< 0.001).

In the first 10 minutes (Figure 2-A), no significant main effect was found for rearing condition (F=1.289, P=0.266) or drug administration (F=0.017, P=0.897), which was accordance with the results in total 60 minutes (Figure 2-B) (F=0.121, P=0.731; F=0.044, P=0.835).

### Effect of NT4-NAP/AAV on the SCT

The relative sucrose preference at baseline showed no statistical differences among four groups before SI started (F=0.387, p=0.763) (Figure 2-C). After SI-rearing and nasal administration, both rearing condition (F=5.932, P=0.031) and drug administration (F=4.414, P=0.045) had main effects on the relative sucrose preference, while no significant interaction effect between them was found (F=1.425, P=0.256). A significant decrease of relative sucrose preference was found in SI mice compared with GH mice (P<0.001), which indicating the core symptom of anhedonia was successfully derived. The administration of NT4-NAP/AAV reversed the deficits of SI mice, with relative sucrose preference almost return to the control values. No apparent change was observed for GH mice between those two administrations (Figure 2-D).

### Effect of NT4-NAP/AAV on the FST

In FST, both rearing condition (F=7.096, P=0.013) and drug administration (F=7.014, P=0.013) had significant effects on the immobility time. But no statistical differences of interaction effect between rearing condition were found (F=0.094, P=0.761). For the empty AAV group, the immobility time of SI mice was significantly longer than that of the GH mice (P< 0.01). The NT4-NAP/AAV treatment decreased the immobility time of SI mice (P< 0.001), which indicating a potential anti-depression efficacy. No apparent change was observed for GH mice between those two administrations. (Figure 2-E).

### Effect of NT4-NAP/AAV on plasma CORT

**Figure 3:**
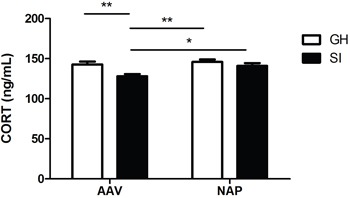
Detection of plasma CORT concentration by ELISA SI mice showed lower plasma CORT concentration than GH mice in AAV group (P<0.01). NT4-NAP/AAV treatment on SI mice reversed CORT decreasing, which nearly returned to GH control levels.

In the detection of plasma CORT, both rearing condition (F=15.420, P=0.001) and drug administration (F=4.918, P=0.035) had main effects on it, while no significant interaction effect between them was found (F=1.253, P=0.273). A significant decrease in concentration of CORT was found in SI mice compared with GH mice (P<0.01). However, the administration of NT4-NAP/AAV reversed the deficits of SI mice, with CORT concentration nearly return to the control values. No apparent change was observed for GH mice between those two administrations. (Figure 3).

### Effect of NT4-NAP/AAV on hippocampus 5-HT

**Figure 4:**
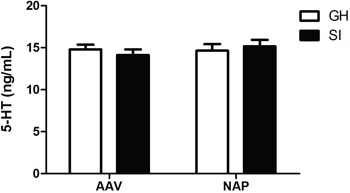
Detection of hippocampal 5-HT concentration by ELISA No statistic differences were found among the four groups.

Neither rearing condition (F=1.354, P=0.255) nor drug administration (F=0.359, P=0.554) had main effcets on concentration of hippocampus 5-HT. No statistic differences were found among the four groups.(Figure 4).

### Effect of NT4-NAP/AAV on the expression of BNDF in hippocampus

**Figure 5:**
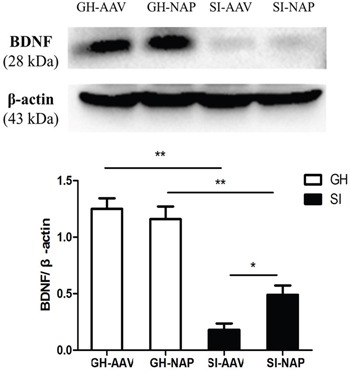
Western-blot analysis for hippocampal BDNF espression Compared with the GH mice, SI mice showed a decreased expression of BDNF in hippocampus (P<0.01). The NT4-NAP/AAV treatment enhanced the BDNF expression in SI mice (P<0.05) but not in GH mice.

As shown in Figure 5, BDNF expression in hippocampus was significantly decreased in SI mice compared with the GH mice (P<0.01). The NT4-NAP/AAV treatment enhanced the expression of BDNF in SI mice (P<0.05), while no apparent change was observed in GH mice (Figure 5).

## DISCUSSION

In postnatal period, the developing brain is sensitive to environmental events. Exposure to early social deprivation, such as child neglect, abandonment or abuse, might induce alterations in the normal development of the neuroendocrine and neurobiological systems, which may lead to increased risks related to dysphrenia disorders, including MDD, schizophrenia and so on [23, 24]. SI represents a lens through which the cellular, neural, hormonal, behavioral, genetic and epigenetic mechanisms of social species could be explored. As early as a quarter century ago, a review of prospective epidemiological studies pointed out that SI was a significant risk factor for morbidity and mortality as obesity, smoking, high blood pressure and sedentary lifestyle [25], which has been proved subsequently by meta-analysis [26]. Experimental researches in nonhuman social species indicate that isolation has direct, detrimental physiological effects. Except for the abnormal emotion [8], recognition and social interaction [8], animal experiments have attested that SI could lead to hyperactive locomotor response in rats [27]; decrease open field activity in pigs [28]; delay neurogenesis in adult rats [29]; and increase the activation of the sympatho-adrenomedullary response to an acute stress in rats [30]. However, it is still controversial that whether SI should be proposed as a valid model for depression.

We investigated the behavioral modification of SI-rearing after weaning. The OFT showed the SI-rearing mice exhibited no difference in locomotion in a novel environment compared with GH mice, which was consistent with previous studies on mice [6, 12, 22]. Under such conditions, the increase of immobility time in the FST was considered as an effective screening test to evaluate the behavioral despair induced by SI-rearing [6]. This depression-like behavior in FST was also found by other researchers [31–33], and it could be reversed by antidepressant [32]. Moreover, the decresed relative sucrose preference derived in SCT has been proposed as a measure of anhedonia, which was an core symptom and corroborative evidence of depression [34].

Even though numerous neurotransmitters and pathways are assumed to be involved in the pathophysiology of depression, the underlying mechanisms for development and progression of depression induced by SI-rearing have not been fully cleared. Three hypotheses of pathogenesis of depression were adopted and validated in the present study, that is, (i) dysregulation of hypothalamic-pituitary-adrenal (HPA) axis, (ii) monoamine insufficient and (iii) hippocampal BDNF insufficient. Little was known about the biological mechanisms underlying the depression-like behavior upon SI-rearing, therefore, we attempted to explore it based on the three hypotheses mentioned above.

Abnormalities in HPA axis function seems to have direct implication on depression. Lots of evidence indicate that HPA axis activity is significantly enhanced in patients or animals suffered from deperssion, compared with the healthy controls [31, 33, 35, 36], while we found the lower plasma CORT concentration in SI mice than GH mice. This discrepancy might be caused by three reasons. Firstly, HPA axis activity could exhibit considerable variability between individuals. In a meta-analysis including 18,454 individuals from 361 studies [35], only 64% of depressed individuals have greater cortisol values than that of non-depressed individuals. Second, HPA axis activity changes depending on other stressor additional to SI-rearing. Basal CORT levels are not altered [37], or even lowered [38] in SI mice housed in standard cages. But it might be elevated by more aversive stressors such as wire floor cages [39], acute restraint stress [31] and forced swimmin stress [33] after SI-rearing. Thirdly, the time of blood sampling, bodily fluid in which the hormone was measured and age of participant were important moderators as well. Besides, a decreased BDNF expression in hippocampus of SI mice was observed, which was accordant with previous studies [40, 41]. Therefore, we hold our opinion that SI-rearing could induce the depression-like behavior, and the possible regulatory mechanism includes dysregulation of CORT and hippocampal BDNF insufficient. However, we failed to found any obvious evidence showed 5-HT dysbolism was involved in this procedure.

Delivering antidepressant into the central nervous system (CNS) is always a challenge because of the impenetrable nature of blood brain barrier (BBB). Intranasal administration is a noninvasive route that provides a direct pathway from nose to CNS via the olfactory apparatus [42]. It was reported that administration of an AAV vector expressing target gene could successfully penetrate olfactory mucosa and lead to enhanced bioavailability of drugs [43, 44]. Although favourable neuroprotective effects of NAP was obtained in plenty of studies, it was not satisfying because of its’ half-life period was only 15 mins’ *in vivo* [45]. Thus, an AAV vector with NAP was desinged and applied in our study. Once administrated intranasally, the AAV vector intruded and colonized into the olfactory mucosa cells, consistently expressing the NAP like a small “perpetual mobile”. This approach has been demonstrated of slower rate of spreading with a time lag to reach the CNS [46]. This intriguingly explains the phenomenon in our present and previous investigation [21, 22] that antidepressant effects of AAV vectors emerged only after a relatively long duration of intranasal administration.

Besides, we have constructed fusion gene NT4-NAP attempting to overcome the challenges that the NAP protein was hard to pass through the cell membrane alone. The nonfunctional prepro region of NT4, a well characterized neuroprotective protein, was attached to the 5′ end of NAP cDNA. On one hand, it made up the lacking of prepro region of NAP and benefited its post-translational modification and secretion [47]. On the other hand, this fusion of NT4 and NAP consisted of an N-linked glycosylation site and a dibasic cleavage site, which ensuring its cleavage intracellularly after peptide translation before its secretion into extracellular space [48]. At this point, we have established a long-lasting secretory system by AAV vector with fusion gene. Once the cells of peripheral tissue were transfected effectively, adequate neuroprotective peptid would be synthesize and secrete, which had the therapeutic effect on neurodegenerative disease.

According to our immunocytochemistry results *in vitro*, the expression of NAP can be successfully observed in PC12 cells infected with NT4-NAP/AAV. As well, the stable and long-term antidepressant efficacy was also confirmed, which has been rarely studied before. Abundant studies amassed a compelling case for a diverse role for NAP against different endogenous and environmental factors [49]. Divinski et al. found NAP was internalized into neurons and neuronal-like cells and co-localized with microtubules [50]. The protein tubulin, constituting the microtubule backbone, was considered as a target of NAP and its related peptides [50]. NAP was also shown to enhance interaction between microtubules and microtubule associated proteins, and the microtubule polymerization [51]. NAP treatment protected microtubules from colchicine by inhibiting hyperphosphorylation of protein tau [52]. Moreover, microtubule is an essential component of the cytoskeleton that supports dendrite and provides platforms for intracellular transport. Dendritic spine pathology is associated with many psychiatric diseases [53–55]. For example, depression is associated with the structural and molecular remodeling of dendritic spines in the hippocampus, prefrontal cortex, amygdala, and nucleus accumbens [55–57]. Antidepressants have reversed some of these structural changes observed in animal models of depression [57, 58]. Therefore, we speculate that the stabilization fortification of microtubules by NAP treatment might play a part in its anti-depression efficacy.

Overall, we have provided evidence that SI procedure appears to be at least as valid as any other animal model of depression. The model has good face validity (phenomenological similarities between the model and patients, including anhedonia, the core symptom of the melancholic subtype of major depressive disorder) and construct validity (the reasonable theoretical rationales such as dysregulation of CORT and hippocampal BDNF insufficient). What's more, the antidepressant effect in SI group was observed after intranasal administration of NT4-NAP/AAV, suggesting that this might be a promising therapeutic strategy for major depressive disorder.

## MATERIALS AND METHODS

### Construction and production of NT4-NAP/AAV

The AAV Helper-Free System (Stratagene) was used for the viral vector preparation. The AAV used in our present study are based on serotype 2. According to sequence of NAP cDNA (TTACGAGGTCAAAGGTAGGGAGTT), the forward primer (5′CGGATCCATGCTCCCTCTCCCCTCATGC3′) and the reverse primer (5′CGGGATCCCTCGAGTCATTGAGGAATGGAAACTGG3′) were designed. Restriction enzyme sites (BamH I and Xho I, at upstream and downstream respectively) were added in the NAP cDNA by PCR. The product was linked to Plasmid pGEM-T Easy which was then transferred into recipient bacterium. The successfully transferred bacterial colony was selected by enzyme identification and was cultured to generate the NAP cDNA with the added restriction enzyme sites. Then NT4-NAP was obtained from pGEM-T Easy/NT4-NAP and linked to vector plasmid pSSCMV. pSSCMV/NT4-NAP was then obtain by alkaline lysis method. Three plasmids (pAAV/Ad, pAAV/Ad cofactor pSSCMV/NT4-NAP) were transfected into HEK293 cells to generate virus NT4-NAP/AAV. NAP expression was confirmed by immunocytochemistry after transfectted on PC12 cells.

### Animals and drug administration

C57 BL/6J mice (aged: 3 weeks; average body weight: 9 ± 2 g) were used for experiment. All the mice were purchased from Vital River Laboratories (Beijing, PR China) and only male mice were used. All experimental procedures were approved by the Animal Care and Use Committee of Xi’an Jiaotong University. All efforts were made to minimize the number of animals used and their suffering.

32 mice were divided into two groups: social isolation (SI) and group housed (GH). For SI group, the 3-week-old mice were bred individually in cages (26×18×13 cm) for six weeks, while GH mice were bred on normal conditions (four per cage) in cages (26×18×13 cm) for six weeks. There were two subsets in both SI and GH group, separately. The first subset underwent behavioral tests (open field test, sucrose consumption test and forced swimming test) and western blot, the second was for enzyme-linked immuno sorbent assay.Finally, there were eight mice in each subset. All the mice were rearing under standard animal housing conditions in 12-hour/12-hour light-dark cycle (lights on at 7:00 A.M.) at a room temperature of 22 ± 1°C and 55 ± 5 % humidity. Mice were given free access to water and food.

NT4-NAP/AAV was delivered quaque die by nasal administration for consecutive 10 days before the beginning of test. The volume of 10 μl (about 8.32 × 10 ^7^ genome copies of AAV vector) for each mouse was applied according to the titer test. Meanwhile, the same dose of empty AAV vector was used as control. The blinding method was applied in the experiment, that is, the nasal administration, operation of behavior test and the analysis of data were conducted by different people individually. This procedure has been described in Materials and methods.

### Open field test (OFT)

Mice were placed individually into an open field chamber (45 × 45 × 45 cm) under a dim light (25 lx) for 1 hour and the tracks were recorded by a video tracking system (SMART, Panlab SL, Barcelona, Spain).

### Sucrose consumption test (SCT)

Mice were deprived of water for 14 h before the test, and the test was carried out in the next morning at 10 a.m. The sucrose was dissolved with distilled water at the concentration of 1% (mass/volume). Mice were provided with one bottle of sucrose solution and another bottle of distilled water during one hour period. Relative sucrose preference= sucrose preference (%)/weight (g). Sucrose preference= sucrose intake/(sucrose intake+ distilled water intake)*100%. The baseline was measured before SI started, and another test was applied after OFT.

### Forced swimming test (FST)

After one day's interval, mice were placed into a Plexiglas barrel (15 cm diameter, 25 cm height) filled with 10 cm of water at room temperature (22 ± 1°C) for 6 min. The mice were dried immediately and returned to the home cage after test. The immobility time was calculated by an observer without knowing the grouping.

### Enzyme-linked immuno-sorbent assay (ELISA)

Blood samples and hippocampus were obtained from the second subset of mice. Plasma corticosterone (CORT) and hippocampus 5-hydroxytryptamine (5-HT) were measured using mouse ELISA Kit (Shanghai Hushang Biotechnology, Shanghai, China) following standard procedures as instruction. The optical density (OD) of the sample was determined at 450 nm using a Metertech microplate reader (BioTek Instruments. Winooski. USA) after the reader was zeroed using the blank well. Then the concentration of each sample was extrapolated from a standard curve. Each determination was done in duplicate. The variation between duplicate values was less than 5%.

### Werstern blot

Hippocampal brain-derived neurotrophic factor (BDNF) was evaluated by western blot. Mice were sacrificed after FST. The hippocampus was isolated on ice. The use of primary antibodies for BDNF (3160-1, 1:1000, rabbit polyclonal, Epitomics) and β-actin (C4, 1:5000, mouse monoclonal, Santa Cruz Biotech) was describled in our previous study detailly. The Quantity One software (Bio-Rad, Hercules, CA, USA) was used for quantification analysis.

### Statistical analysis

All data were expressed as mean ± standard error of mean (SEM), using SPSS13.0 software for data processing and analysis. The data normally distributed were analyzed using two-way analysis of variance (ANOVA) with rearing condition and drug administration as between-subject factors. ANOVA was conducted among the four groups, followed by a LSD post hoc test. P values < 0.05 were considered to be statistically significant.
